# Flexibility Support for Homecare Applications Based on Models and Multi-Agent Technology

**DOI:** 10.3390/s151229899

**Published:** 2015-12-17

**Authors:** Aintzane Armentia, Unai Gangoiti, Rafael Priego, Elisabet Estévez, Marga Marcos

**Affiliations:** 1 Automatic Control & Systems Engineering Department, ETSI Bilbao, University of the Basque Country (UPV/EHU), 48013 Bilbao, Spain; unai.gangoiti@ehu.eus (U.G.); rafael.priego@ehu.eus (R.P.); marga.marcos@ehu.eus (M.M.); 2 Electronic and Automation Engineering Department, University of Jaen (UJA), 23071 Jaén, Spain; eestevez@ujaen.es

**Keywords:** AAL systems, homecare, adaptability, availability, stateful components, domain modeling, multi-agent systems

## Abstract

In developed countries, public health systems are under pressure due to the increasing percentage of population over 65. In this context, homecare based on ambient intelligence technology seems to be a suitable solution to allow elderly people to continue to enjoy the comforts of home and help optimize medical resources. Thus, current technological developments make it possible to build complex homecare applications that demand, among others, flexibility mechanisms for being able to evolve as context does (adaptability), as well as avoiding service disruptions in the case of node failure (availability). The solution proposed in this paper copes with these flexibility requirements through the whole life-cycle of the target applications: from design phase to runtime. The proposed domain modeling approach allows medical staff to design customized applications, taking into account the adaptability needs. It also guides software developers during system implementation. The application execution is managed by a multi-agent based middleware, making it possible to meet adaptation requirements, assuring at the same time the availability of the system even for stateful applications.

## 1. Introduction

Developed countries are suffering a demographic change as a result of the growing number of older people as well as an increase in longevity [1,2,3]. Elderly suffer from typical age-related diseases demanding expensive medical care that is pressuring public health systems. Governments, conscious of this problem, are funding research projects aiming at providing new ways of medical care [4,5,6,7]. Indeed, issues like extending healthy life expectancy, improving quality of life, and maintaining autonomy and independence, are part of the term “active ageing” that was adopted by the World Health Organization in the late 1990s [8]. In this context, home-based care solutions seem useful to provide personalized care, improve comfort, autonomy, confidence and safety of the residents, optimizing, at the same time, medical resources [2,9,10].

During the last years, Ambient Assisted Living (AAL) systems have emerged as an adequate technological support for elderly and disabled to enhance their quality of life avoiding social isolation [11,12,13,14,15]. AAL systems have been studied by several authors with different purposes, from energy efficiency or comfort optimization to dealing with safety or recognizing elderly activity [16,17,18,19,20,21,22], as well as for home care [11,14]. In the particular case of home care for elderly, smart homes are equipped with sensors, actuators and other appliances, whereas patients are provided with medical sensors and medical staff with personal computers, mobile phones, or PDAs. The captured data are analyzed in order to be aware of the continuous evolution of the patients and the environment, as well as for early detection of alarming situations (alarm triggering). This is also the case in homecare applications where mechanisms to define and process the sensing and processing of biomedical and environmental signals are needed. However, sometimes a simple alarm warning might not be enough, and flexibility to evolve as patient status and its environment do is also necessary, indeed often without direct external intervention (adaptability). This might imply starting new applications, stopping, or even modifying existing ones. Thus, to achieve the goal of adaptable monitoring of elderly, applications must be context-aware being able to modify their behavior according to changes on their context.

Besides, these applications are commonly executed in distributed and heterogeneous environments, and mechanisms for managing widespread and specific devices with different capabilities (from embedded devices to those with high processing capacities) are necessary. As they supervise the health of patients, their response must be efficient in order to react as quickly as possible to dangerous situations, so a suitable resource management system is needed, not only because it is essential for dealing with the limitations of embedded systems, but also to ensure efficiency. Preventing service disruptions is also mandatory in order to avoid information losses, especially in emergency cases. Consequently, availability must be guaranteed in case of failure in processing nodes or sensor devices. Finally, other critical aspects are privacy, confidentiality and integrity of the data about patients (safety and security).

Therefore, AAL systems for the elderly raise several challenges for developers that have to be taken into account at the requirements analysis and design phases, and that have to be ensured at runtime [23,24,25]. There are several works that deal with safety, privacy and security issues related to data storage, processing and transmission. Message encryption using Public Key Infrastructure (PKI) and Secure Socket Layer (SSL) [26], authorization and authentication mechanisms [27,28], and the development of security frameworks [29,30] or safety policies [31] are the most usual solutions.

On the other hand, there are also middleware systems that help an application to interact or communicate with other applications or hardware through networks. These kinds of middleware systems are commonly built over a framework layer which solves ubiquity challenges. Examples of such frameworks are Open Services Gateway Initiative (OSGi) [32], Remote Procedure Call (RPC) [33], Object Request Broker (ORB) [34], Reflection [35] or Foundation for Intelligent Physical Agents (FIPA) [36] compliant frameworks.

Self-adaptive systems are commonly defined in the literature as those capable of automatically modifying themselves in response to changes in their operating environment [37]. This requires self-awareness and context-awareness, *i.e.*, the system must be aware of its own state by means of monitoring both, existing resources and its context. Nevertheless, most of them are ad-hoc solutions that assume stateless applications and, as far as authors know, they do not offer means for defining the application evolution to context changes.

This paper focuses on these issues, adaptability and availability, identifying the needs of the target applications and offering appropriate mechanisms to meet both requirements at the different phases of the application life cycle. In particular, mechanisms for defining, based on the medical expertise, how the application must evolve to context changes as well as mechanisms to manage the application at run-time, assuring that the application is available in case of device failure even for stateful applications. This latter is achieved by means of a multi-agent based middleware (MAS).

Previous works of authors are related to applying modeling techniques for developing service-based applications without the necessity of a central orchestrator [38,39]. Additionally, the preliminary idea of the multi-agent based middleware proposed in this work was presented in [40]. With respect to these previous works, this paper contributes a domain modeling approach that allows systems definition from different points of view. The user view (medical staff) defines, using concepts from the area of expertise, the monitoring of patients and their environment, including the adaptation of the applications to context changes (patient or environment). The software view guides the software developer in the design and implementation of the components required for providing the medical care specified in the user view. The paper also extends the preliminary middleware to manage events signaling special situations and the associated actions to be taken. Finally, application availability is assured by taking advantage of the mobile nature of agents. This is a generic approach as the middleware offers generic agent templates to be used to define any application that evolves with its context.

The remainder of this paper is as follows: Section 2 presents some related work on both adaptability and availability in home care AAL systems. Section 3 identifies the challenging requirements demanded by homecare applications. This section also includes a brief description of the proposed solution that consists of a domain modeling approach and a MAS middleware. In Section 4 the modeling approach for defining the application dynamic behavior is presented while Section 5 presents the MAS-RECON middleware that provides a set of agent types for implementing flexible homecare applications as well as mechanisms to manage their execution. Section 6 is dedicated to the assessment of the proposal based on the implementation of a healthcare demonstrator and some experimental tests. Finally, Section 7 outlines the most important conclusions and future work.

## 2. Related Work

This section comprises some research work dealing with the focus of the paper, *i.e.*, adaptability and availability in homecare applications for elderly.

As far as authors know, the majority of works in the literature lack *adaptability* mechanisms, as they focus on alarm triggering in case of danger, asking for medical assistance or warning the patient. In this context, some works provide closed solutions that can be configured by the final user such as [41]. Other works provide means for application design aiming at alarm identification [28,42,43,44,45,46] or at the specification of the responses [47,48], which commonly correspond to warning or alarm triggering. For instance, the Millennium Home System [47] allows defining how to select the best mode of interaction with the user, and whether the resident or an external service have to be warned. In this context, one of the easiest ways of covering a broad range of situations and responses is the use of the event-condition-action (ECA) paradigm [49]. How ECA rules allow defining the actions that have to be executed when certain events are detected is presented in [48].

With respect to alarm identification there exist different approaches in the literature. For example, the CommonSens system [42] proposes an event language to describe events, and the Necesity project [43] presents a rule-based classifier that determines if a situation is normal or abnormal. Some authors make use of modeling methodologies [50] as they allow representing a system at different abstraction levels, hiding irrelevant technical details [28,44,45]. In this sense, the specification and verification approach in [44] combines UML diagrams and formal methods for establishing time requirements associated to events. These design approaches have something in common; they focus on software developers. On the contrary, but also based on modeling techniques, there are works that incorporate domain experts in the system design and development as in [45] where physicians define the conditions to trigger the alerts to display in a view, or in [28] where they model the care process and nurses manually initiate the different actions related to an alarm. The CAALYX system [46] proposes a special purpose language for caretakers to define the clinical rules. These rules detect health alteration by means of observation templates that are customized for patients in the so-called observation patterns. Among the analyzed works, the CAALYX system might be the most similar to the work presented in this paper. However, as far as authors know, it is neither possible to automatically start the execution of new observation patterns as a result of an alert (dynamic adaptation), or to relate changes on the environment with the monitoring of patients.

Related to implementation issues, there is a substantial body of literature on self-adaptive systems based on reconfigurable middleware systems. As previously stated, this kind of middleware systems are commonly built over a framework layer which solves ubiquity challenges simplifying component management, update and communication.

The THOMAS middleware [27] combines multi-agent technology and service orientation, offering registration mechanisms for services, their implementations and organizations. It allows the organizational structure to be dynamically modifying by creating new ones, or by adding and removing members. However, this capability is restricted to some concrete roles. In the CARISMA project [35], self-adaptation is tackled by defining profiles as fixed sets of actions the middleware should take when a specific event happens. Another approach is based on the so-called sentient objects [51] which are able to take decisions and perform actions. Actions to be performed as a response to context changes can be statically defined as part of the middleware at design time [52], or they can be built at runtime [53,54]. Nevertheless, these approaches are not fully generic as they are presented as part of an application domain and therefore they represent an ad hoc solution to a concrete problem. On the contrary, the iLAND project [55] proposes a general-purpose middleware for real-time systems with time-bounded reconfiguration capabilities. However, it only supports sequential stateless applications and it does not provide support for managing context events.

Application *availability* even in case of device failure is usually managed at the application level, and therefore the application state is implicitly managed by itself. This is the case of the architectures defined in [27,56] offering redundant service providers. The service oriented component model described in [57] provides location independent peer to peer (P2P) communications between components. The GAL platform [58] also defines services as reusable blocks and availability is assured by means of redundancy on services. In the iLAND middleware [55], availability is supported by creating several implementations related to a service. Therefore, the formers present application aware recovery and the latter only supports stateless services recuperation in case of a node failure.

## 3. Flexibility Requirements for Home Care AAL Systems

For a better understanding of the requirements identified in this section, the next paragraphs describe some use cases related to an old people’s home. These use cases have been documented in and inspired by some literature works related to heart rate, blood pressure, oxygen saturation and body temperature monitoring [59,60,61,62,63]. They illustrate simple examples of real use cases being simple enough to represent the flexibility demands of this type of applications.

*Use Case 1 (UC1)—Body temperature monitoring*. After a surgical operation, the body temperature of a patient is measured four times a day. These values are stored for further analysis. Additionally, if the temperature is over a concrete threshold (according to the patient particularities), the medical staff has to be warned in order to supervise a possible infection.

*Use Case 2 (UC2)−Heart rate monitoring*. In order to detect a possible heart attack, the pulse rate of a patient is monitored every 10 min. However, if the heart rate trend indicates an abnormal increase (according to the patient particularities), apart from warning the medical staff, the acquisition frequency must be increased for a more detailed monitoring.

*Use Case 3 (UC3)—Fire detection*. In a nursing home, monitoring the physical environment is crucial. In case of fire, collecting information about health of patients might help emergency services to make decisions on arrival. Thus, buildings are usually equipped with fire detectors and upon the detection of a fire new health monitoring tasks must be launched for every patient, such as pulse rate and oxygen saturation level monitoring.

*Use Case 4 (UC4)—Blood pressure monitoring*. The main objective of this use case is to monitor the blood pressure of a patient, four times a day. However, as it is presented in [62], blood pressure measures are only relevant if the patient is relaxed. This situation can be checked by means of its pulse rate. Therefore, before taking a blood pressure reading it is necessary to supervise the pulse rate of the patient (one measure every 30 s) until it is relaxed or a maximum waiting time is exceeded. Of course, if the pulse rate or the blood pressure is out of range, medical staff has to be warned.

As it can be concluded from these examples, target applications have three main objectives: (1) monitoring; (2) early recognition; and (3) rapid and suitable reaction. To match these goals, context information is captured by means of sensors that monitor vital functions (all use cases) and acquire environmental measures (UC4), taking into account that each measurement must be performed at the right frequency (temperature is taken every six hours in UC1 whereas heart rate is measure every 10 min in UC2). The processing of these data enables a continuous monitoring of the patient health and their environment, being possible to foresee risky situations and to provide the most suitable assistance in case of emergency. Furthermore, actions for acquiring biomedical sensor measurements and related processing must be customized for every particular elder, although there are similarities among many of them. Indeed, there exist medical guidelines that give support to medical professionals in making general decisions on the treatment of a patient, which, in the end, varies from patient to patient. For instance, pulse measurement can be always carried out in the same manner, whilst a concrete pulse value has a different meaning according to various factors such as the patient age, physical activity, ambient temperature, *etc.*

Target applications supervise dynamic systems that can evolve to dangerous situations. For instance, the pulse rate of a patient increases in case of heart attack. In these situations, besides the usual monitoring and alarm detection, a reaction to the alarm must be defined. Warning medical staff, as in UC1, is the easiest response which is already performed by the works described in the related work section. However, sometimes the application has to evolve in response to relevant changes on its context. As a result, it could be necessary to change the acquisition rate as in UC2 (in case of a possible heart attack), or to initiate the processing of new biomedical values as it is stated in UC3 (new monitoring tasks have to be launched after fire detection) and UC4 (blood pressure monitoring is started). Sometimes, as in UC4 it is necessary to stop current actions (heart rate monitoring is stopped after patient is relaxed).

Finally, continuous monitoring implies to assure application availability even in case of node failure. Furthermore, service recovery has to be application unaware, that is, the application design has not to be altered to match this requirement. Special attention has to be paid to the particular case of those services whose result depends on previous executions (the so-called stateful services). For example, in the UC4 (blood pressure monitoring), several subsequent pulse rates must be analyzed in order to assure the relaxed condition. When a node executing this analysis fails, the recovery process implies restoring the previous pulse rate values. In summary, the main requirements demanded by the target applications are collected in Table 1.

**Table 1:** Requirements demanded by the target applications and their relation with the proposed use cases.

Requirement Identifier	Requirement Description	Use Cases that Represent It
R1 Personalized sensing and processing	Support for different sensors, customized processing and thresholds.	All
R2 Distributed and heterogeneous environments	Integration of distributed sensors and heterogeneous platforms (resources).	All
R3 Activation and execution types	Actions triggered by time or by event.	All
R4 Adaptability	Context changes awareness: modifying timing properties, launching/stopping applications…	UC2, UC3 and UC4
R5 Availability	If a device fails, application must remain unaffected.	All

In order to meet the requirements identified above, this paper proposes to divide a monitoring action into a set of measuring and processing tasks that are customized according to the particular health problems of the patient. These tasks can be executed in distributed and heterogeneous devices and have to be interconnected to achieve the monitoring goal. With this purpose, this paper proposes a system architecture that consists of a domain modeling approach and a multi-agent based middleware, the so-called MAS-RECON middleware.

The domain modeling approach guides the specification of applications and the implementation of the corresponding components. It has two stakeholders, medical professionals and software developers, and therefore it has been divided in two domains: the user view and the software view. The definition of the user view is the responsibility of the medical professionals as they define the customized treatment for every patient. It consists of a set of interconnected tasks in order to provide the required medical service, which includes the monitoring, alarming situation detection and reaction. On the other hand, software developers are the responsible for the software view which is based on the previous one. It comprises the set of components in charge of acquiring and processing the biomedical and environmental signals. These components are connected following the logic established by the medical staff. Therefore, software developers implement the required medical services and the application logic to connect them. In a sense, the modeling approach allows the medical professionals to specify the software developers what to do and how to do it, by using concepts close to its area of expertise.

The MAS-RECON middleware extends the Java Agent DEvelopment (JADE) framework [64] and manages the execution of applications. In order to implement the application code, software developers are provided with code templates that have to be filled in with the functional specification of the user view. At runtime, the proposed middleware architecture together with the logic added to the code templates and the negotiation capabilities of agents are the means to support the flexibility of applications and fault tolerance. The next sections detail the proposed domain modeling approach and middleware architecture.

## 4. Domain Modeling Approach for Application Specification

In order to reach a correct and full-customized health monitoring, it is necessary to incorporate domain experts in the system definition and development. With this purpose, this section describes a domain modeling approach that allows defining the whole application abstracting the implementation issues. As previously mentioned, two different but related domains have been identified: the user view and the software view. More precisely, the software view generalizes the user view by extending existing concepts with new properties and by adding new concepts.

### 4.1. User View

Medical professionals and maintenance staff provide the information related to the *user view* that is constituted by a set of concepts and relationships among them. These concepts allow defining the functional requirements (R1, R2 (Table 1)), the timing requirements (R3) and the dynamism (R4) needed for the health monitoring of patients and the supervision of the environment, from the medical professionals perspective. Availability requirement (R5) is application unaware and thus, it is not covered by the modeling.

#### 4.1.1. Functional Requirements (R1, R2)

**Figure 1:**
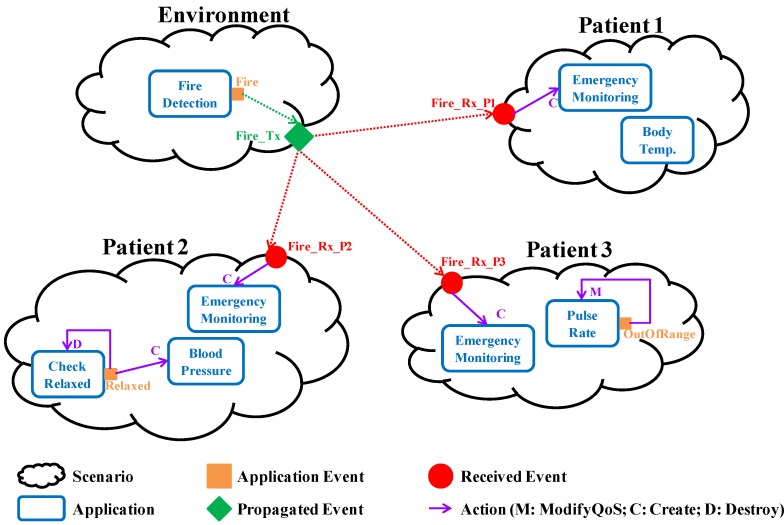
Graphical representation of the user view specification related to a nursing home with three patients.

This view defines the health monitoring of every patient and the supervision of the environment by means of the *Scenario* concept. Figure 1 illustrates the concept of Scenario through the specification of a nursing home (*System* concept) with three patients (Scenarios).

Health monitoring has to be customized to each patient. Therefore, physicians have to identify which biomedical variables to monitor and how to process them, using the *Application* concept. For example, as it is depicted in Figure 1, the special monitoring for emergency situations described in UC3 is defined for every patient. “Patient 1” represents a resident without any relevant health problem. Its temperature is monitored as it has been operated on. “Patient 2” is related to a resident with hypertension. Thus, its blood pressure has to be controlled as it is explained in UC4. Finally, “Patient 3” refers to a resident with heart disease (UC2).

The monitoring of a biomedical or environmental variables involves several tasks (*AppComponent* concept), including: data acquisition at concrete frequencies from sensors (or extracted from a repository in bulk); and processing activities to obtain useful data for the medical staff. Medical professionals have to describe these tasks (name and providedServiceDesc properties) from which software developers implement the needed software components (again, AppComponent concept). For example, in order to check the body temperature, four tasks are necessary: one for temperature acquisition (once a day), another for storing these measures in a repository, other one to check if the captured values are out of the normal range of the resident, and the last one to warn the medical staff in case of detecting an abnormal situation.

#### 4.1.2. Timing Requirements (R3)

Non-Functional requirements are collected as properties related to the previous concepts. Indeed, specifying the timing requirements of every monitoring is essential (timingProps of the Application concept). More precisely, it is necessary to identify when the monitoring has to be activated (activation properties) and how it has to be performed after activation (execution properties). For example, in the case of “Patient 2” blood pressure has to be measured four times a day which implies that it has to be periodically activated every 6 h. However, after activation its pulse rate has to be periodically monitored every 30 s until relaxing (periodic execution), whereas blood pressure is measured just once (one-shot execution). Additionally, there are also configuration parameters (configParam property) such as the patient identifier that allow the customization of the health and environment monitoring.

#### 4.1.3. Adaptability (R4)

Lastly, the *Event* concept allows medical professionals and maintenance staff to identify relevant context changes that demand a reaction. Therefore, they have to detail how to detect every relevant situation and how to react to them. Note that, as it is collected in [65], the context term may have very different meanings. In this work, it refers to the health status of a patient and/or the state of the physical environment. Detecting a context change is the result of data processing. For example, in the heart rate monitoring described in UC2, an abnormal increase of the heartbeat is considered a relevant context change (OutOfRange event in Figure 1). In the same manner, it is essential for UC3 specifying under which circumstances the captured environmental signals (smoke, temperature, *etc.*) are related to a fire (Fire event in Figure 1). In UC4, the instant at which the patient relaxes is relevant (Relaxed event in Figure 1). This is detected after processing several pulse rates, between its maximum heart rate (HR_max_) and its resting heart rate (HR_rest_).

On the other hand, specifying how to react against a relevant context change comprises the actions to be performed after its detection (*Action* concept). There are several types of actions and every one refers to an Application, the target of the action from now on. For instance, in UC2, after detecting a risky situation, it is necessary to increase the acquisition frequency (Modify action). Therefore, the target of the action is the monitoring itself. In Figure 1 this fact is represented by a purpled line that starts on the OutOfRange event and which points to the monitoring itself. However, as it is also depicted in Figure 1, once a patient is relaxed (*i.e.*, the Relaxed event is triggered) the actions are to finish pulse rate monitoring (Destroy action) and to start blood pressure monitoring (Create action). Note that in these examples, after detecting a context change of a patient, the actions performed are related to monitoring tasks of the same patient. But sometimes the actions drawn from a context change goes beyond the patient itself, that is, context changes are propagated (*TxScnEvent* concept). This is the case of the fire detection that requires the starting of a particular emergency monitoring for all the patients at the nursing home, as it is illustrated in Figure 1 (Fire_Tx). Therefore, a scenario can propagate events and it can also receive events propagated by other scenarios (*RxScnEvent* concept). The latter also has associated actions whose target application belongs to the scenario itself.

### 4.2. Software View

**Figure 2:**
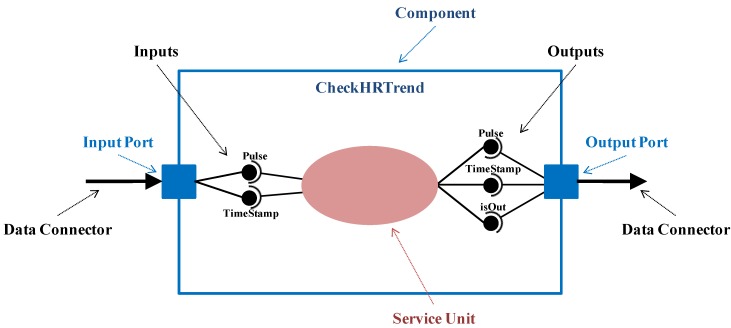
Detailed representation of the CheckHRTrend component.

The software view inherits the user view and extends it to define the tasks specified by the medical professionals. In this context, an Application is defined as a set of components (AppComponent concept) that cooperate to achieve application tasks (R1, R2). Therefore, at the software view an application component represents a set of monitoring activities (service unit, from now on), together with the application logic (which data has to be sent, when and to which components) and the event-triggering logic (detection of relevant context changes and reaction), previously defined at the user view. A service unit requires a set of input parameters to offer its service and it provides a set of output parameters after its execution. From now on, component parameters and service unit parameters are interchangeably used (*Parameter* concept). Figure 2 illustrates the CheckHRTrend component belonging to UC2. It detects if the heart rate evolves to exceed the normal range of the “Patient 3” (isOut parameter). It requires a pulse value (Pulse parameter) and the time instant (TimeStamp parameter).

Application components are also characterized by the timing properties and configuration parameters (R3). For example, a periodic pulse rate monitoring means that the component in charge of the sensor reading has to be periodically executed, but other components will be executed on demand, *i.e.*, after data reception. Additionally, it is also necessary to indicate if the service unit requires additional initialization or finalization actions, and if its execution depends on the result of previous executions (stateful component).

Application components cooperate by exchanging all the data necessary to provide their service, *i.e.*, by connecting the input parameters of a component with the output parameters of its predecessors. With this purpose, components are provided with an input port (*InputPort* concept) and/or an output port (*OutputPort* concept), linked through connectors that collect the exchanged data (*DataConnector* concept). Thus, ports encapsulate the interactions with the service unit and with other components.

**Figure 3:**
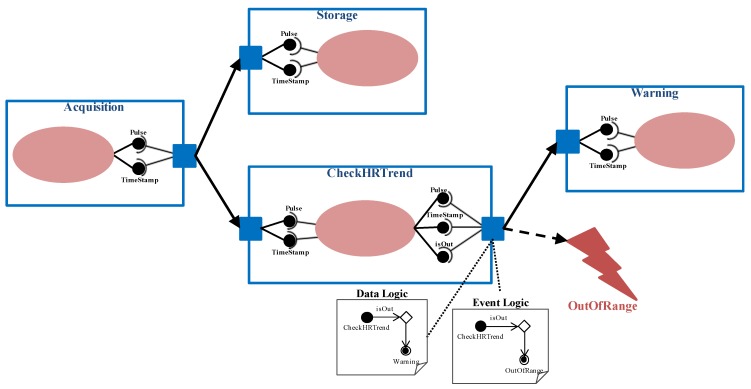
Definition of the application for heart rate monitoring.

More precisely, the input port is in charge of receiving data from predecessors, providing the service unit with the necessary input parameters. Similarly, the output port collects the output parameters resulting from the service unit execution, delivering them to the follower components. Every input parameter received by an input port through a data connector has a peer connection with the corresponding output parameter sent by an output port through it. It is important to remark that software developers have to check that both data-types are compatible in order to set these connections (*DataConnection* concept). The CheckHRTrend component depicted in Figure 2 has an input port to receive its inputs through the incoming data connector whose source is the Acquisition component, as it is illustrated in Figure 3. Similarly, it has an output port to send part of the provided output parameters to a subsequent component, through an outgoing data connector whose target is the Warning component. Data connections are established between the output parameters of the Acquisition component and the input parameters of the CheckHRTrend component, as well as between the input parameters of the Warning component and the output parameters of the CheckHRTrend component.

Cooperation among components can be leaded by certain logic, which implies that interface compatibility must be considered from a global point of view. In particular, two different output logic types have been identified (logic property related to the OutputPort concept): Default and Customized. The Default logic implies that the outputs of the service unit are always sent to all followers. In the example of Figure 3, the acquired pulse values are always sent to be stored (Storage component) and to be analyzed (CheckHRTrend component).

In order to take into account other possible cases, for example when data are delivered under a condition, the customized logic has been defined. This logic is represented by the *DataLogic* concept that is expressed by means of a UML activity diagram associated to the output port. The ‘Initial Node’ corresponds to the current component. “Control flows” are based on expressions containing output parameters of the component. And every “Activity Final Node” refers to a subsequent component. In the example presented in Figure 3, when heart rate tendency is abnormal, medical staff is warned through the Warning component. This logic is depicted in the “Data Logic” activity diagram attached to the output port of the CheckHRTrend component.

All the concepts introduced up to now allow expressing the R1, R2 and R3 requirements. On the one hand, they enable the definition of health monitoring customized to patients with the associated timing properties. On the other hand, combining measurements and processing is met as applications are decomposed in components that can be executed in different nodes.

In order to consider the adaptability needs derived from relevant context changes (R4 requirement), the proposed software view extends the user view founded on the idea of the ECA rules. It provides mechanisms for defining how to detect a relevant context change and how to react to it, following the specifications of medical professionals. The detection of context changes is part of the processing (service unit) a component performs, giving the result in any of its output parameters. Thus, the component has an event port (*EventPort* concept) with an activity diagram associated, represented by the *EventLogic* concept. This activity diagram is similar to the one for data logic, but in this case the “Activity Final Node” represents the event to trigger when a context change is detected. In Figure 3, there is an “Event Logic” diagram associated to the event port of the CheckHRTrend component. It represents that a risky trend of the heart rate triggers the OutOfRange event.

The Action concept that represents the actions triggered by events has been also extended. In the Create action, some of the new application components can be started with an initial execution state which is obtained from the execution state of a component of the current application (stateInfo property). In the Modifiy action the new timing properties of the application have to be indicated. Note that some of these actions must be executed following a concrete order (sequence property) whereas others can be executed as decided by the middleware.

### 4.3. Meta-Model

**Figure 4:**
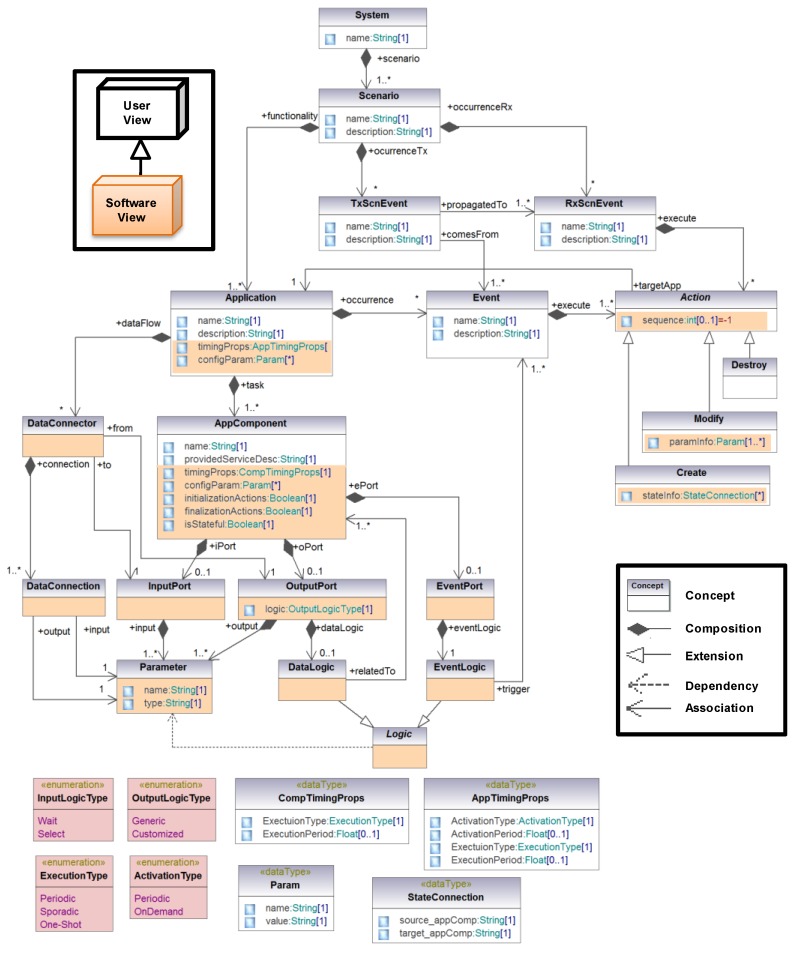
Meta-model of the domain modeling approach for application specification.

All the concepts described in the previous sections, as well as the relationships and restrictions among, them are presented in Figure 4. Concepts are depicted by means of rectangles. Relationships are classified into four groups: (1) composition (black diamond). For example, a scenario for a patient is composed by a set of health monitoring applications; (2) extension (white arrow). For instance, the Create action extends the abstract Action by adding new properties; and (3) dependency, to state that a concept must be aware of another. For example, the logic for event triggering (EventLogic) is based on the output parameters of the application component; and (4) association, to reference other concepts. For instance, actions are associated to applications. Finally, restrictions are represented by means of the multiplicity associated to the relationships among concepts.

## 5. MAS-RECON Middleware

This section presents the MAS-RECON middleware, a multi-agent based middleware in charge of managing the execution of homecare applications for the elderly modeled in the previous section. The domain modeling approach allows medical professionals to specify the functionality of these applications and their adaptability needs to evolve to context changes. Therefore, the middleware must provide mechanisms for implementing the functionality (meeting R1, R2, R3 requirements), by managing the execution (synchronous and on demand) and communication of application components. It must also provide flexibility mechanisms to enable the adaptation at runtime (R4 requirement). This is met through the event concept implementation, and to assure application unaware availability in case of node failure (R5 requirement. In particular, the proposed availability mechanism is based on a negotiation process among the nodes for finding the most suitable node to hold a new instance of a failed component.

**Figure 5:**
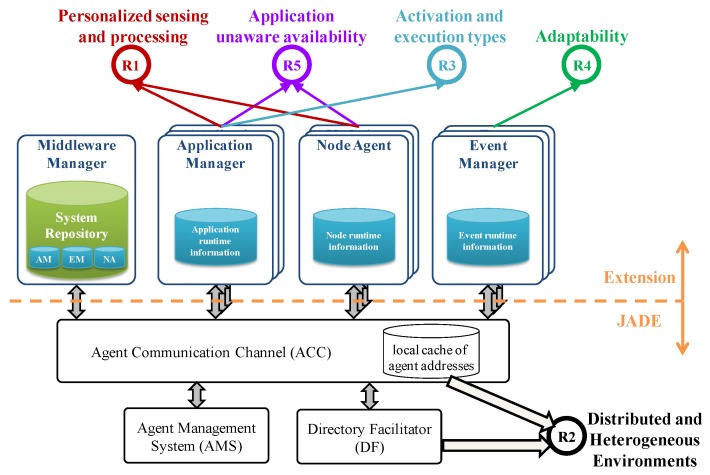
Architecture of the multi-agent based middleware.

Taking all these demands into account, Figure 5 depicts the proposed middleware architecture founded on the JADE framework. JADE is a software framework that facilitates the development of interoperable intelligent multi-agent systems. The JADE framework has been extended with the new modules depicted at the upper part of Figure 5 in order to meet the requirements identified in Section 3. (1) a Middleware Manager (MM) which is the main system orchestrator; (2) an Application Manager (AM) module per application, in charge of managing the life-cycle of its components as well as their execution state; (3) a Node Agent (NA) module per node that provides runtime information about the node that is useful for availability support; (4) an Event Manager (EM) module per event that manages all its related actions. Each middleware module is implemented by an agent running in the multi-agent system.

### 5.1. Functional and Timing Requirements (R1, R2 and R3)

The JADE framework is a FIPA compliant agent framework fully developed in the Java programming language. The FIPA foundation promotes agent-based technology and the interoperability of the FIPA standard with other technologies. A FIPA compliant infrastructure must support agent management by means of the following modules (see bottom part of Figure 5): the Directory Facilitator (DF), the Agent Management System (AMS), and the Agent Communication Channel (ACC). According to the FIPA specification, there must be at least one DF agent in the platform, which supplies the yellow pages where agents can register offered services or look for required services. The AMS manages the agent creation, removal and migration. The ACC supports interoperability within and across different platforms. Finally, the so-called Internal Platform Message Transport (IPMT) provides a message routing service for agents on a particular platform.

The domain modeling approach allows distribution as applications are defined as sets of interconnected components that comprise the provided service, the logic to connect them and the logic for event triggering. In this context, the underlying JADE framework allows fulfilling the R2 requirement (distributed and heterogeneous environments) as every component instance is an agent running on the system and agents are mobile in nature. Additionally, as Java is platform independent and JADE can run even in embedded devices, the proposed middleware supports different types of nodes with different capabilities, from embedded devices such as mobile phones and sensors to those with high processing capacities.

Additionally, as these distributed agents cooperate by exchanging messages, three FIPA compliant ontologies have been defined in order to support communications among agents: (1) Data ontology for message exchange containing the data necessary to provide a medical service or environment supervision, such as sensor values and processing results; (2) Command ontology for control commands that allow agents or technicians interact with the middleware modules and vice versa; (3) State ontology for updating the execution state of an agent (value of relevant variables).

**Figure 6:**
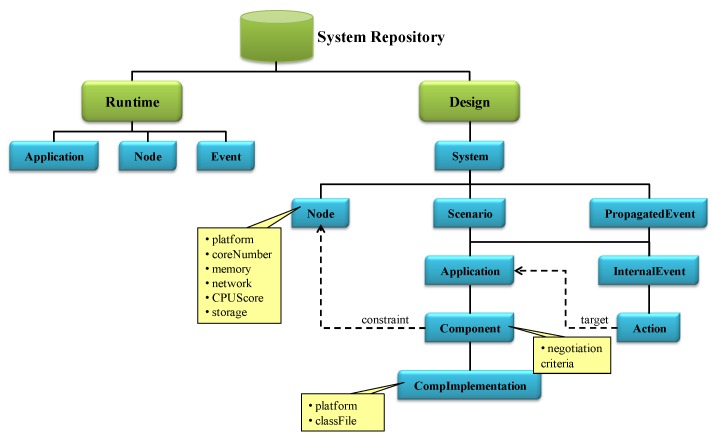
Structure of the System Repository at the Middleware Manager (MM) module.

The MM module manages information about the whole system which is collected in the so-called System Repository. The hierarchical structure of this repository is presented in Figure 6. It contains runtime information about the running applications, the triggered events and booted nodes. This part of the repository is distributed throughout the corresponding middleware modules. It also contains design information including the physical nodes and the data coming from the software view.

Physical nodes are the hardware devices where the instances of application components run. This includes access to sensors, actuators and processing units. Every node contains an instance of the NA module which provides physical information (core number, storage and memory capacity, network speed, CPU score and platform) and runtime information (CPU usage, free memory) about the node. A NA registers itself automatically at boot time, providing the MM with its resource capabilities (highlighted in yellow at Figure 6). They also perform the negotiation process when it is required by the AM.

Software developers are the responsible for registering the information related to the application itself: the system, the scenarios, the applications that belong to each scenario and their components. Events and the actions to be performed have to be also registered. Two types of events have been considered: internal events (InternalEvent) and propagated events (PropagatedEvent). Internal events belong to a scenario, and their actions refer to applications of the same scenario: Event concept in Figure 4 and the events received by the scenario and that have been propagated by other ones (RxScnEvent concept in Figure 4). In the middleware, the events propagated among scenarios (TxScnEvent concept in Figure 4) are composed by the set of internal events associated. For example, in the nursing home system depicted by Figure 1, the FireTx event is a propagated event, whereas OutOfRange, Relaxed, FireRx_P1, FireRx_P2 and FireRx_P3 are internal events.

**Figure 7:**
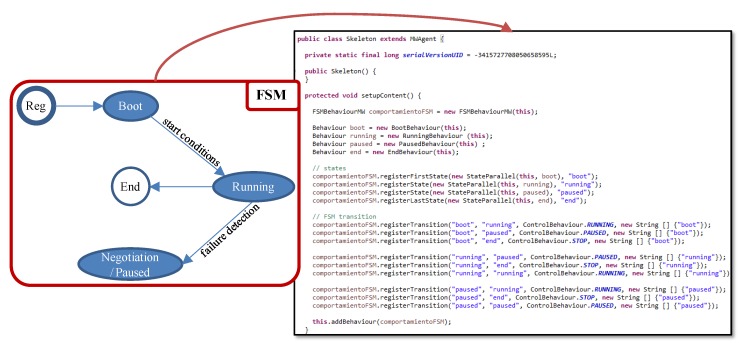
Finite State Machine (FSM) and its Java implementation.

Additionally, the structure of the System Repository takes into account that a component can be implemented in several ways (CompImplementation). Note that it is also possible to restrict the nodes where the instance of a component implementation, component instance or agent from now on, can be executed (constraint). For instance, the software developer may use different platforms or libraries, restricting the available nodes to execute them The need of a concrete sensor only accessible from a node is another example of constraint. This way a task of the user view is linked to a specific node, through a component of the software view. It is important to remark that when adding a new node to the system new component constraints should be registered, if needed. Moreover, the skeleton code of these component instances has been fixed in order to match the R1 (personalized sensing and processing) and R4 (application unaware availability) requirements. More precisely, every component instance must implement the FSM represented in the left part of Figure 7, having the following states:

- *Boot*: during this FSM state, the agent waits until its start conditions are met. This allows executing the required initialization actions and synchronized start of agents. When the start conditions are met, the agent switches to the Running FSM state.
- *Running*: in this FSM state, the agent is offering its functionality related to a medical service. Besides, every cycle the execution state is stored at the corresponding AM, for availability purposes.
- *Negotiation*/*Paused*: when a component failure is detected, the AM forces the agent to this FSM state .
- *End:* during this FSM state the agent finishes its execution which includes the required finalization actions.

The skeleton code derived from this FSM and implemented in Java is also depicted in the right part of Figure 7. Additionally, application components are provided with a control interface through which they receive control commands. The software developer has to customize this skeleton code for every application component founded on the software view of the modeling approach. In particular, if the component requires initialization actions a new Java class that extends the Boot FSM state by including all the needed actions has to be implemented. For example, the pulse oximeter sensor used in the demonstrator must be initialized. Similarly, if the component requires finalization actions a new Java class that extends the End FSM state with these actions has to be implemented. The Running FSM state has to be always customized in order to include the medical service offered, the data logic and the event triggering logic. Therefore, another Java class has to be developed. With this purpose, two templates have been defined according to the activation mode of the agents (R3 requirement):

(1) Periodic: this template is based on the TickerBehaviour class of JADE. It is used for components that execute the service periodically. Therefore, every cycle they run their functionality, send results, if any, update the execution state and delay until the next activation.
(2) On demand: this template is based on the CyclicBehaviour class of JADE. It is used for components that execute the service after the reception of a data message. Therefore, they wait for all incoming messages, run the functionality, send results, if any, and update the execution state.

For example, in UC2 depicted in Figure 3, the component in charge of the pulse reading is executed every 10 min (periodic), but the component in charge of warning the medical staff is executed just after receiving input parameters (on demand).

**Figure 8:**
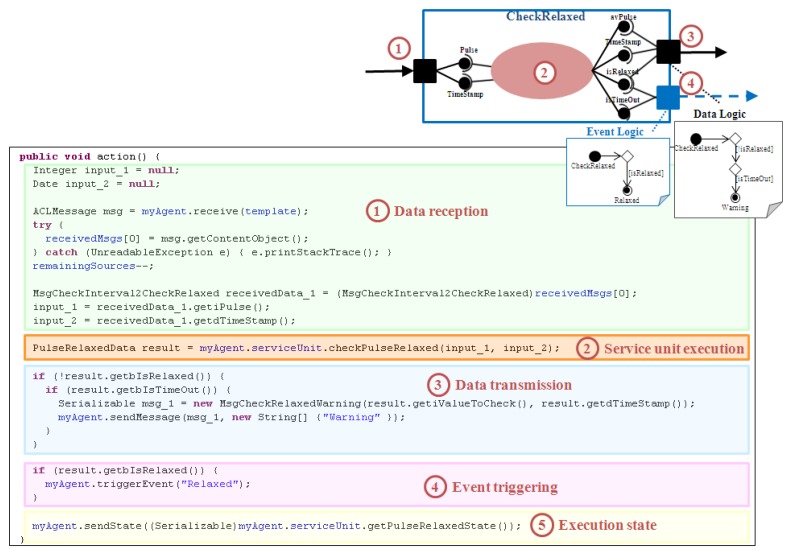
Customization process of the Running FSM state for the CheckRelaxed component.

As an example, Figure 8 presents the customization of the Running FSM state related to the CheckRelaxed component. This component belongs to the Check Relaxed application (UC4, see Figure 1). It receives two input parameters, a pulse value (Pulse) and the measurement instant (TimeStamp). It analyzes the new value together with several previous ones in order to determine if the patient is relaxed or not. Therefore, it provides four output parameters: the average pulse (avPulse), the last measurement instant (TimeStamp), a flag for patient relaxation (isRelaxed), and a flag that indicates if the waiting time has been exceeded (isTimeOut). When the patient relaxes it triggers the Relaxed event (event logic). When the waiting time is over, medical staff is warned (data logic). In summary, the customization process of the Running FSM state comprises the following steps:

(1) If the component has an Input Port, code for data reception has to be included. Every Data Connector of the modeling approach that ends in the input port is related to a data message received from a previous component. The required input parameters have to be extracted from these messages according to the input logic type defined and the Data Connections established.
(2) The code for service unit execution is always added. It depends on how the software developer has implemented this functionality.
(3) If the component has an Output Port, code for data transmission has to be inserted. Every Data Connector of the modeling approach that starts in the output port is related to a data message sent to a subsequent component. The output parameters obtained as a result of the service unit execution have to be grouped according to the Data Connections established, composing all the necessary output messages. Additionally, if the output part has a Data Logic attached, the associated activity diagram has to be parsed in order to write the necessary conditional statements for data delivery.
(4) If the component has an Event Port, the code for event triggering has to be added. Similarly, the associated activity diagram has to be parsed to include the conditions that have to be filled to trigger every event. If the event is propagated through scenarios the event included in this code is the corresponding TxScnEvent.
(5) If it is a stateful component, the code for updating its execution state at the corresponding AM has to be included.

At runtime, the execution of the instances of these developed components is managed by the AM module. The MM deploys as many AM instances as launched applications. Each AM is in charge of supervising the execution of the components associated to an application (R3 requirement). This includes several tasks:

- Components startup, which consists of selecting the appropriate node to hold the component instance, by means of a negotiation process.
- Management of the execution state related to stateful components.
- Management of the component life-cycle. It is aware of the current FSM state of every component instance, and it may force it to pass to a concrete FSM state, if necessary.
- Management of component failure detection, due to a node failure, for example.

### 5.2. Adaptability (R4)

**Figure 9:**
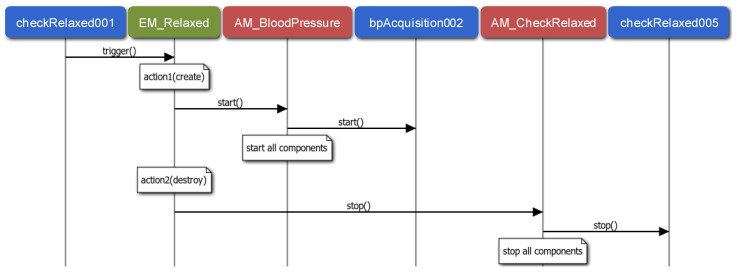
Sequence diagram related to the Relaxed internal event.

In order to tackle the adaptability needs (R4 requirement), the events registered in the System Repository are supervised by an EM module. The MM deploys an EM instance for each event. It performs the actions established for the event and it supervises they follow the required order, if necessary. Note that the interaction between a component instance and the EM is through method invocations at the source code. For instance, Figure 9 presents how the EM related to the Relaxed event (depicted in Figure 1) supervises its associated actions. When the checkRelaxed001 component instance detects that the patient is relaxed, it triggers the Relaxed event. This event triggers two actions: one for launching the blood pressure monitoring and the other one for stopping the pulse rate monitoring, both through the corresponding AM. As an example, the figure shows how the AM of Blood Pressure application (AM_BloodPressure) starts one of its component instances (bAcquisition002), and how the AM of the *Check Relaxed* application stops one of its component instances (checkRelaxed005).

**Figure 10:**
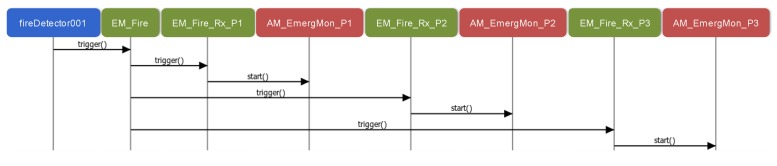
Sequence diagram related to the Fire propagated event.

Similarly, Figure 10 describes how propagated events are attended. In this case, the fireDetector001 component instance detects a fire and thus, it triggers the Fire event. This event is generated in the Environment scenario and propagated to the other three scenarios. As a result, three internal events are triggered, Fire_Rx_P1, Fire_Rx_P2 and Fire_Rx_P3 (see Figure 1), each triggering a Create action for launching the emergency monitoring of the corresponding patient. As it is illustrated in the figure, applications are started through the corresponding AM as in Figure 9.

The benefits of the event manager module are twofold. On the one hand, it optimizes the use of system resources, as upon an event triggering an invocation to the middleware is issued in order to create/destroy/modify applications which in the end implies allocating/de-allocating the corresponding resources. As a result, resources are allocated just when needed. On the other hand, the domain modeling approach provides application independency within scenarios as they are only related through events. Scenarios independence is also supported as they can be connected through propagated and/or received events. At runtime, the EM module implements this independence by executing the actions related to the triggered event. As a result, adding new monitoring applications or adding a new scenario (a new patient) to an already running system does not modify the implementation of the system. Instead, the system extension implies registering the new applications/scenarios and the corresponding events, if necessary, as well as the implementation of the new components.

### 5.3. Application Unaware Availability for Stateful Applications (R5)

**Figure 11:**
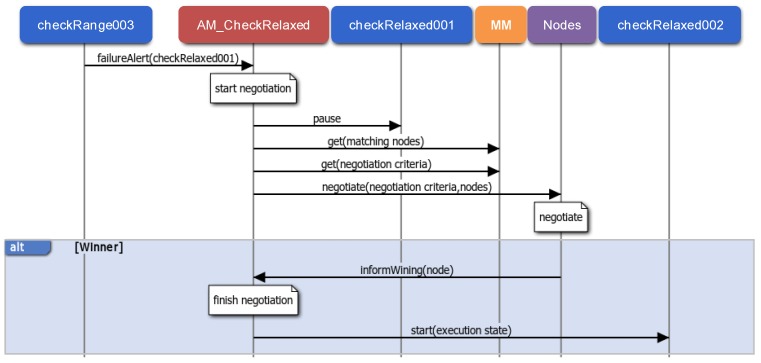
Application unaware availability: failure detection and stateful recovery.

AMs and NAs are the main participants of the middleware support for application unaware availability (R5 requirement). As commented above, the proposed availability mechanism is based on finding the most suitable node to hold a new instance of a failed component. Therefore, on the one hand it is necessary to detect component failures, and on the other hand it is necessary to recover it. As an example, Figure 11 illustrates the recovery of a stateful component. In particular, it is the CheckRelaxed component of the Check Relaxed application (it monitors pulse rate, UC4), whose previous component is the so-called CheckRange.

Component failures can be detected in two ways: when the sender of a data message detects that it has not been possible to deliver it, or when a periodic component exceeds the period to refresh its execution state in the AM. In both cases the corresponding AM is notified and the failed component instance is labeled as faulty. This avoids attending to the same failure more than once. After, the AM starts the recovery process. In the example of Figure 11, the checkRange003 component instance detects a component failure as the data message sent to the checkRelaxed001 component instance has not been delivered.

A component recovery starts with a negotiation among all the NAs related to nodes that can hold a new instance. These nodes are selected taking into account the node constraints and the available implementations of the component, and they negotiate according to the negotiation criteria established during the registration. The negotiation criteria can be, for example, the highest free memory or the lowest processor usage. Once there is a winner NA, the AM finishes the negotiation process, and deploys a new component instance on the winning node, initialized with the last execution state. In the example, as a result of the negotiation process the checkRelaxed002 component instance is started with the last execution state updated by the failed checRelaxed001 component instance.

## 6. Assessment

This section presents the feasibility of the proposed solution in order to cope with the demands of homecare applications, through its feasibility to deal with the requirements identified. On the one hand, the proposal design is validated by means of a homecare demonstrator. On the other hand, its runtime performance is evaluated by means of a set of experimental tests. More precisely, these tests aim at evaluating the adaptability and availability mechanisms offered by the MAS-RECON middleware. Finally, the main benefits and limitations are highlighted.

### 6.1. Homecare Demonstrator

A homecare demonstrator that includes the proposed use cases has been implemented, namely, the nursing home represented in Figure 1. Therefore, there are three residents: Patient 1 has no serious health problems; Patient 2 suffers from high blood pressure, so s/he requires blood pressure supervision four times a day; Patient 3 suffers from heart disease, so s/he is provided with continuous heart rate monitoring (every 10 min). On the other hand, the building is equipped with a fire detection system based on the temperature and CO_2_ concentration.

From the specification point of view, it is a system composed of four scenarios: three patients and the environment. The Environment scenario consists of an application for fire detection that triggers the Fire event, if detected. This event is propagated to the other three scenarios. Therefore, all the patient scenarios receive a propagated event that launches a concrete application for health monitoring in emergency situations (Emergency Monitoring). In the Patient 2 scenario, the Check Relaxed application triggers the Relaxed event when the patient is relaxed, launching the blood pressure monitoring (Blood Pressure application) and stopping itself. In the Patient 3 scenario, there is an application for pulse rate monitoring.

**Figure 12:**
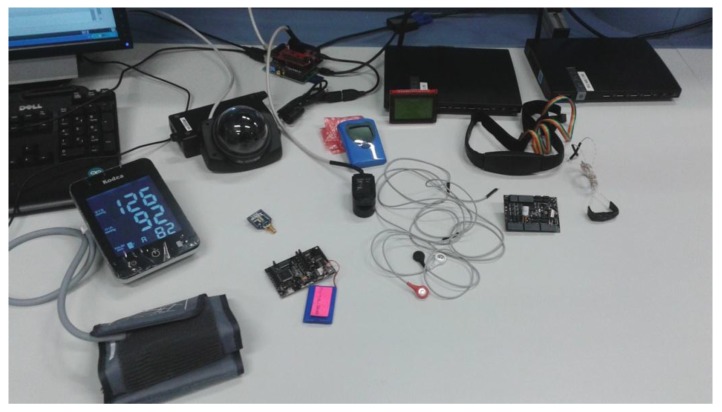
Infrastructure of the healthcare demonstrator: *e*-Health Sensor Platform V2.0., gas sensors kit and processing units.

The prototype demonstrator consists of biomedical and environmental sensors, and processing units. For health monitoring purposes, the biometric shield for Arduino and Raspberry Pi, the so-called e-Health Sensor Platform V2.0., was used [66] (see Figure 12). Every patient is provided with a health sensor shield mounted over a Raspberry Pi. More precisely, it offers a body temperature sensor, a pulsyoximeter (SPO_2_) for pulse rate, and a sphygmomanometer for blood pressure. The environment supervision is performed through temperature and CO_2_ sensors mounted over a waspmote [67]. The processing tasks can be executed in four PCs.

From the implementation and deployment point of view, all the application components have been developed in Java programming language. There is a repository for recording information about patients such as personal data (identifier, name, surname, age, sex…) and medical data according to their health problems. For example, Patient 3 is characterized by her/his maximum heart rate (HR_max_), its resting heart rate (HR_rest_), and its normal range of body temperature. Furthermore, it also stores the historic measures of patients. The patient repository has been implemented by means of the native XML eXist database [68]. The MM, the AM instances and the EM instances run in the same PC. Agents related to application components that manage biomedical sensors are restricted to the corresponding Raspberry Pi. Finally, for availability purposes, agents related to the other application components can be deployed in any of the four PCs.

Taking into account that the use cases illustrate all the requirements identified in Section 3, this homecare demonstrator allows:

- Validation of the Domain Modeling Approach presented in Section 4 as every use case has been designed and developed following it. Note that in this homecare demonstrator there are neither real patients nor medical professionals involved.
- Assessing the middleware architecture design and the services it offers: adaptability to context changes (event management), availability (failure detection and negotiation-based recovery), stateful component management, and registration (system repository).

### 6.2. Runtime Performance

Runtime performance has been assessed regarding the two main goals of the paper: adaptability and availability. In particular, adaptability is evaluated in terms of the reaction time to adapt to a context change (a change on the health status or environment conditions) whereas availability is assessed according to the recovery time under a failure.

Both parameters are tested by using similar experiments. The starting point is a very simple and sequential application that captures a sensor value, processes it and shows the result. In both cases the number of available nodes to hold component instances is incremented. For availability tests the number of processing tasks is also increased, *i.e.*, the number of components of the application. However, for adaptability, the number of actions triggered by the event is increased. In order to avoid that the different processing capacities of nodes interfere the analysis of the results, and taking into account that in a real scenario there are many devices with limited resources, all the nodes in the experiment are Raspberry Pi.

**Figure 13:**
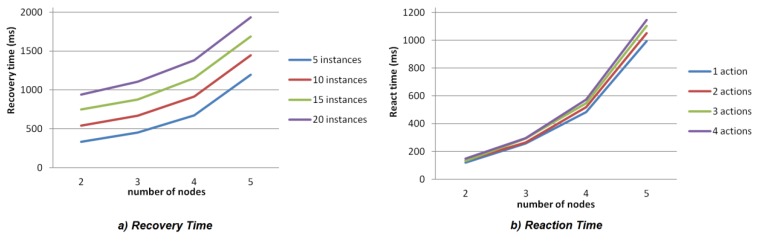
(**a**) Availability metrics: recovery time; (**b**) Adaptability metrics: reaction time.

Regarding availability, Figure 13 shows the recovery time of the different tests. This time ranges from a node failure to the recovery of all the affected component instances. As expected, the recovery time increases with the number of nodes and components. In fact, the recovery time increases almost proportionally to the number of nodes, as more nodes participate in the negotiation and due to the low processing capacities of the Raspberry Pi, this handicaps the negotiation processes. Similarly, recovery time also augments with the number of application components when more instances are affected by the node failure. However, taking into account that the worst case is about 2 s and that the most restrictive application evolves at 30 s (Check Relaxed application), the time delay is acceptable if compared with the benefits achieved. Additionally, this worst case corresponds to applications whose component instances can run in five different nodes, which is unusual as two available nodes are commonly enough.

As far as adaptability is concerned, Figure 13 depicts the reaction time in milliseconds since an event is triggered until all its associated actions have been performed. For simplicity, all the actions triggered by the event are Create actions. Therefore, the resulting time includes the startup of the applications. Again, as expected, the number of nodes and the number of actions increase the reaction time.

**Figure 14:**
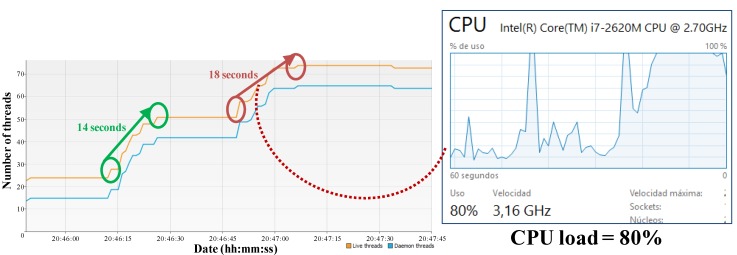
CPU load *vs.* application start time.

In order to identify availability limitations another test has been performed in a PC. The same application of eight components has been created twice: the first time under normal conditions of CPU load, and the second time after significantly increment the CPU load (up to 80%). As a result, the availability performance has been negatively affected. Figure 14 depicts the number of threads in the node (every running agent is a thread). As it is observed, the start time for the same application increases about a 28% because negotiations among nodes are slower. These metrics have been captured by means of the VisualVM GPL software.

**Figure 15:**
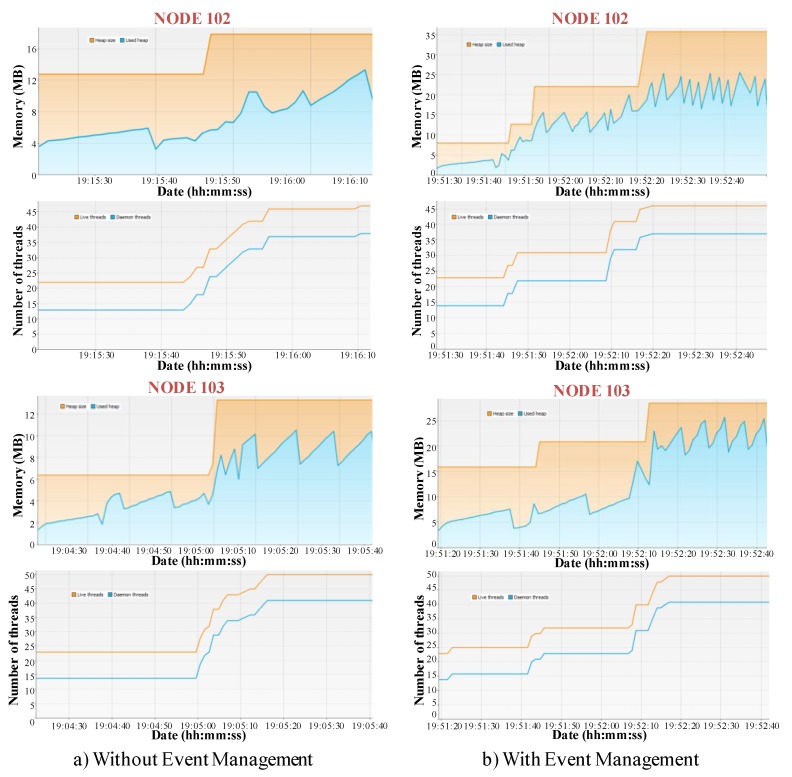
Resource usage in terms of memory usage. (**a**) without event management; (**b**) with event management.

As it has been previously stated, resource optimization is one of the main benefits of the event management. In this sense, resource consumption in terms of memory load has been analyzed by means of an application (composed by 18 components) that, after detecting a relevant context change, creates other five applications of 18 components. The component instances of these applications are deployed in four nodes. Figure 15 compares two different tests (related to two of the available nodes). In every graphic, the upper part represents the memory consumption, the orange line refers to the Java Virtual Machine (JVM) heap whereas the blue line is related to the memory used by the loaded objects (here, the JVM garbage collector activations to free memory are noticed). The bottom part depicts the number of threads on the node:

(a) Without event management: The six applications are started from the beginning. When the first application detects the context change, it sends a data message to the first component of the rest applications in order to activate them. This implies that memory resources are allocated from the start. As a result, in both nodes the amount of memory does not change after the start.
(b) With event management: the first application triggers an event that is managed by an EM module that performs five Create actions. In this case, there is an initial amount of memory allocated, and after the event triggering, the amount of allocated memory increases.

These metrics prove that events management improves resource usage, which is very useful when resources are limited. However, it implies more reaction time as application components are started after event triggering. More precisely, when there is no event management, reacting to an event by means of an application creation just involves the synchronized start of all the application components, as all have already executed the needed initialization actions.

These tests also show the good performance of negotiation mechanisms. In fact, as the negotiation criterion is the “highest free memory”, all the component instances are similarly distributed among the available nodes. This is showed in the bottom part of the graphics in Figure 15.

## 7. Conclusions and Future Work

This paper presents a solution for the design, implementation and management of homecare applications for elderly. The proposed system architecture consists of a domain modeling approach and a multi-agent based middleware and it provides mechanisms to tackle their flexibility demands to adapt their behavior according to changes on their context (patient health status or environment conditions) and to avoid service disruption.

The use of domain modeling techniques allows defining applications from different points of views, each gathering the information relevant to it. As a result, the proposed modeling approach allows medical staff to design a personalized monitoring of the health status of patients and environmental conditions. It takes into account adaptability needs from the design phase as it is possible to identify relevant context changes, defining how to detect and how to react to them (user view). Additionally, it guides software developers in the implementation of all the needed software components which contain not only the medical service execution but also the logic for data exchange and the logic for event triggering (software view).

At runtime, multi-agent technology has been adopted to convert components into intelligent entities. In this context, the proposed MAS-RECON middleware has extended the JADE framework in order to manage the execution of these applications, providing mechanisms that allow performing adaptation and that assure availability even for stateful applications. More precisely, the Event Manager module controls all the actions related to an event. As a result, an optimized resource usage is achieved. Availability is assured by recovering the execution of the failed component instance in the most suitable node. This is possible due to the failure detection, stateful recovery and negotiation mechanisms provided by the Application Manager and the Node Agent modules.

The feasibility of the proposal has been proved by means of a healthcare demonstrator based on a nursing home. Several representative use cases have been identified and implemented. Experimental results show that recovery time (availability) and reaction time (adaptability) are affected when the number of nodes that can hold component instances increase or when the number of actions triggered by an event increases. Furthermore, supporting adaptability and availability implies an extra time that is acceptable if compared with the benefits achieved: maintaining application state and resource optimization.

However, the middleware architecture does not support fault tolerance. For example, if an AM fails, the runtime data and execution state related to its application components are lost. The middleware lacks of admission control mechanisms to assure that enough resources are available as the system grows. Therefore, further work is aimed at exploring the distribution of the system repository for improving fault tolerance of the middleware modules, and implementing the admission control. Additionally, proactive mechanisms will be also added in order to match Quality of Service (QoS) parameters. For example, load balancing mechanisms for achieving energy efficiency at node level, or unbalancing mechanisms for energy efficiency at system level (using the least number of nodes). Additionally, as it has been proved in the assessment section, limited resources decrease the middleware performance due to slower negotiation actions. Thus, future work is also focused on supporting flexible QoS for non-critical applications. Finally, Model Driven Engineering techniques will be explored as they allow automating application design and the code generation process.
